# Variability in Journal and Publisher Policies on Artificial Intelligence Use in Manuscript Preparation: An Orthopaedic Perspective

**DOI:** 10.2106/JBJS.OA.26.00221

**Published:** 2026-09-09

**Authors:** Nicholas Kelly, Jillian Neuner, Sanjeev Sabharwal

**Affiliations:** 1 Department of Orthopaedic Surgery, University of California, San Francisco, San Francisco, California

## Abstract

**Background:**

Artificial intelligence (AI)-generated text has been detected in nearly 90% of orthopaedic peer-reviewed publications, yet public disclosure of AI use remains extremely low. Whether journal and publisher policies for authors on AI use are present, sufficiently detailed, and internally consistent has not been systematically examined in orthopaedics. We characterized the prevalence, scope, and within-publisher consistency of AI policies across a structured sample of leading orthopaedic and biomedical journals.

**Methods:**

We performed a cross-sectional analysis of publicly available author instructions, editorial policies, and publisher guidance for 32 journals (6 core orthopaedic, 19 orthopaedic subspecialty, 7 general biomedical) across 12 biomedical publishers. Forty-one variables were extracted per journal between March and April 2026, including presence of policy for AI use for authors, disclosure requirements, and use of AI for data analysis, generating figures and references, and policies for peer reviewers. A second reviewer independently coded a 12-variable subset for 10 journals (31% calibration) to assess inter-rater reliability (Cohen's kappa = 0.90, 96% agreement).

**Results:**

Twenty-nine of 32 journals (91%) had an identifiable AI policy for authors; 3 subspecialty journals had none. All core orthopaedic (6/6) and general biomedical (7/7) journals had policies, versus 16 of 19 subspecialty journals (84%). Among journals with policies, 28 of 29 (97%) required disclosure of AI use by authors and 22 of 29 (76%) prohibited listing AI as an author. Coverage of other domains was less frequent: figures and images (66%), hallucinations or fabrication (62%), AI-generated references (48%), peer review (45%), and data analysis (41%).

**Conclusions:**

Domains that can directly impact scientific integrity and clinical practice, such as AI-assisted manipulation of data, statistical outputs, fabricated references, and undisclosed image alterations, are currently not consistently queried in detail at manuscript submission. With advances in software technology, biomedical journals can test and adopt AI-detection tools that are both sensitive and specific to screen all submissions before peer-review to maintain scientific integrity and transparency.

**Level of Evidence:**

Level IV, Cross-Sectional Study.

## Introduction

The availability of large language models such as ChatGPT has impacted several facets of biomedical publishing, including orthopaedics. Sweeney et al. reported artificial intelligence (AI)–generated content in 89.8% of 196 articles across 10 leading orthopaedic journals, yet only 1.0% had publicly disclosed AI use despite all 10 journals requiring such disclosure^1^. Similar patterns of AI use by the authors have been noted in orthopaedics and other biomedical fields^2-5^. As AI-detection tools combine high sensitivity with meaningful false-positive rates on heavily edited prose^6,7^, the true prevalence of AI use by the authors may be varied and lower than estimates suggest. Furthermore, part of the apparent gap in disclosure by the authors may reflect lack of clarity about what constitutes reportable “AI use” rather than deliberate concealment by the authors. Even so, the reported gap between likely use and documented disclosure appears substantial.

Part of the challenge for stakeholders, including the authors, editors, and publishers, is that “AI use” spans a wide array of applications, from grammar editing, to drafting and literature synthesis; to generating figures, references, or analyses; to peer-review assistance; and, at the far end, complete fabrication of scientific content. These varied applications of AI use by the authors carry different associated risks to scientific and ethical integrity and clinical care. For instance, language polishing that leaves the scientific content unchanged has substantially different implications compared with a fabricated citation or falsified image without acknowledgement. Journal policies treating all AI use as equivalent, with a single act of disclosure, may be poorly matched to the degree of harm that most threatens scientific integrity.

The International Committee of Medical Journal Editors, the World Association of Medical Editors, and the Committee on Publication Ethics have suggested guidelines that state that AI tools cannot meet authorship criteria and that AI use must be disclosed at submission^8-10^. Biomedical journals including a joint editorial from some of the leading orthopaedic journals have offered further insight regarding the use of AI by the authors^11-13^. However, translation of these guidelines into specific, unambiguous, and readily accessible journal policies and instructions to the authors has been inconsistent. Cross-disciplinary analyses have noted inconsistency in biomedical journal policies for the use of different AI applications by the authors^14-16^, but to our knowledge, no prior study has specifically examined the variability of instructions to the authors for AI use across a sample of orthopaedic journals.

Building on the reported prevalence of AI-generated content in orthopaedic publications^1^, we asked a complementary question: Are AI policies coherent, consistent, and detailed enough across a sample of orthopaedic journals an author may encounter? While this is not meant to be an in-depth analysis, we characterized the prevalence, scope, and within-publisher consistency of AI policies for the authors across a structured sample of leading orthopaedic and biomedical journals. Because these policies change rapidly, our findings are a snapshot as of March-April 2026 that may have since changed.

## Methods

We performed a cross-sectional analysis of publicly available Instructions for Authors (IFA), editorial policies, and publisher guidance for 32 journals across 12 publishers: 6 core orthopaedic journals (the flagship general journals of the major orthopaedic societies), 19 subspecialty journals (by 2025 Google Scholar h5-index), and 7 high-impact general (nonorthopaedic) biomedical journals included as comparators rather than the analytic focus. Our journal sample was partly based on the leading orthopaedic titles examined by Sweeney et al.^1^ One reviewer extracted 41 variables in the context of AI use for manuscript preparation and review per journal (Supplementary Appendix 1) between March and April 2026, including policy presence, disclosure requirements, and treatment of AI authorship, figures, peer review, data analysis, and references. A second reviewer independently coded a 12-variable subset for 10 journals (31% calibration; Cohen's κ = 0.90, 96% agreement). Strictness, centralized publisher policy, and within-publisher consistency were coded (Table I and Supplementary Table 1 footnotes), with borderline cases assigned to the journal’s most frequent article type (e.g., original research vs. review or commentary). As our observations used publicly available information and did not involve human subjects, it was exempt from IRB approval.

**Table I:** Publisher-Level AI Policy Characteristics (n = 32 Journals Across 12 Publishers)

Publisher	Journals (n)	Has AI Policy	Centralized Publisher Policy?	Strictness Mix Among Sampled Journals	Within-Publisher Consistency
Elsevier	9	9 (100%)	Yes	9 moderate	Identical
Wolters Kluwer	8	7 (88%)	No	1 strict, 6 moderate, 1 none	Variable
Wiley	4	3 (75%)	Yes	3 moderate, 1 none	Variable
SAGE	2	2 (100%)	Yes	2 moderate	Identical
Springer Nature	2	2 (100%)	Yes	2 moderate	Identical
Single-journal publishers*	7	6 (86%)	—	6 moderate, 1 none	N/A
All publishers	32	29 (91%)	—	1 strict, 28 moderate, 3 none	—

Strictness was coded as *strict* (AI use prohibited or restricted to specific manuscript components only), *moderate* (AI permitted with disclosure requirements), or *none* (no AI policy identified). For tiered policies, the modal article type was coded. A centralized publisher policy denotes a single AI policy applied across a publisher’s journals; within-publisher consistency reflects whether sampled journals from the same publisher displayed the same policy (Identical, Variable, or Not applicable for single-journal publishers).

AI = artificial intelligence.

* Single-journal publishers in our sample: BES (*BJJ*), ACP (*Annals of Internal Medicine*), AMA (*JAMA*), BMJ Publishing Group (*BMJ*), Massachusetts Medical Society (*NEJM*), PLOS (*PLOS Medicine*), and Thieme (*Journal of Knee Surgery*).

## Results

Twenty-nine of 32 journals (91%) listed an AI use policy for manuscript preparation on their website. For context, Ganjavi et al. found only 24% of their sampled journals had such policies in October 2023^14^. In our sample, all core orthopaedic (6/6) and general biomedical (7/7) journals had some stated AI policies, compared with 16 of 19 subspecialty journals (84%). One biomedical journal listed a detailed AI policy on a separate publisher’s website that was absent from its own journal submission guidelines.

Among journals with AI-related policies for the authors, 28 of 29 (97%) required disclosure of AI use by the authors (a yes/no determination of whether any disclosure was mandated) and 22 of 29 (76%) explicitly prohibited listing AI as an author. The recommended location within the manuscript for where the authors should disclose the use of AI varied widely, including the Methods section (11 journals) or a separate disclosure section (4), with others requiring such statements in the cover letter, multiple locations, or none, underscoring the inconsistency the authors may face with even this simple requirement.

**Fig. 1:**
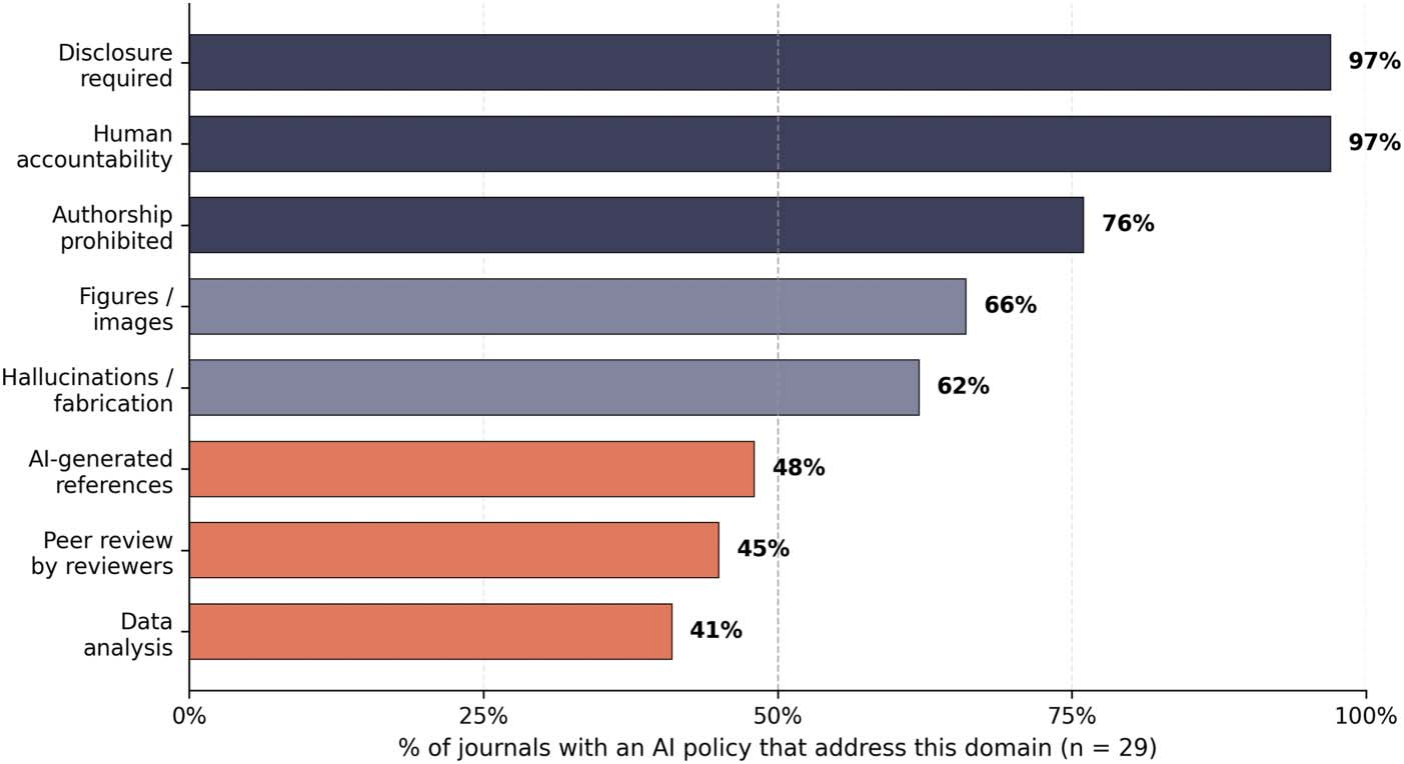
Proportion of journals with an AI policy (n = 29) that explicitly address each policy domain. Domains most directly tied to research integrity—verification of AI-generated references, peer-review confidentiality, and data analysis (shown in orange)—were the least frequently addressed, whereas disclosure, human accountability, and authorship (dark) were nearly universal. The dashed line marks 50% coverage. AI = artificial intelligence.

Detailed disclosure requirements varied substantially across different domains (Fig. 1). Disclosure of AI use (97%), human accountability for checking accuracy of AI-assisted content (97%), and AI-authorship exclusion (76%) was addressed by most journals. Explicit policies for the use of AI were less common for domains tied to research integrity, such as the use of AI for generating figures and images (66%), flagging AI-generated content that is factually incorrect or fabricated (62%), verifying AI-generated references (48%), use of AI for peer review (45%), and data analysis (41%).

**Fig. 2:**
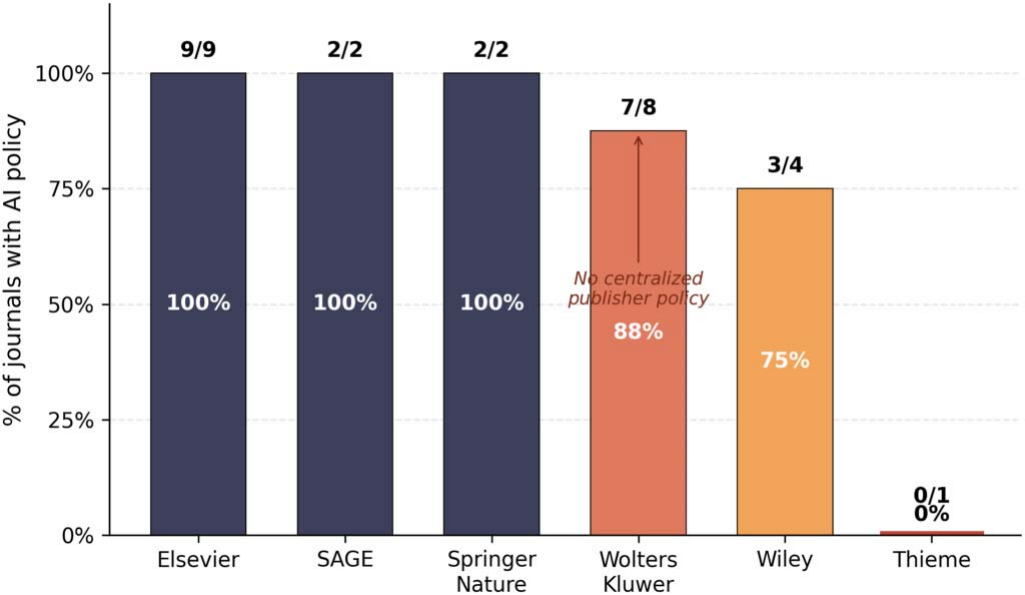
Proportion of sampled journals with an identifiable AI policy, by multijournal publisher (n = 5 publishers). The single multijournal publisher without a centralized AI policy showed the greatest within-publisher variability. Single-journal publishers are not shown, as within-publisher consistency cannot be assessed from a single journal; their aggregate adoption was 6 of 7 (86%). AI = artificial intelligence.

Variability in AI policies was often greatest among journals from the same publisher (Table I, Fig. 2). Three publishers applied an identical centralized template across all sampled journals within their portfolio, whereas 1 publisher without a centralized policy showed the widest range, from a highly detailed policy to none for the journals supported by them.

Two further observations illustrate this variability in AI-related policies among sampled journals. One journal that had migrated between publishers still displayed its former publisher's AI policy, inconsistent with its current publisher. Another applied article-type–specific rules atop its publisher's policy, with stricter guidelines for the authors of commentary than research content, as reflected in a general medical journal’s tiered AI policy^17^.

## Discussion

Our observations have a direct bearing on the disclosure gap recently reported by Sweeney et al.^1^ A reported 90% prevalence with only 1% disclosure is unlikely to solely reflect deliberate noncompliance by the authors. Besides the potential limitations of the emerging AI detection tools such as excessive sensitivity leading to false positives, ambiguous, and inconsistent guidelines among journals and publishers regarding the use of AI possibly contributed to the previously reported findings^1^. We also noted that several of the less-addressed domains such as fabricated references, AI-introduced errors or alterations in study data, and undisclosed image manipulation are among those most likely to damage scientific integrity, whereas the most consistently mentioned policy, AI authorship, is largely symbolic since an AI tool cannot assume ultimate responsibility for a manuscript. Our findings align with those of Perkins and Roe, who identified inconsistent scope and enforcement as a central weakness of publisher AI guidance^15^.

Based on our review, we offer suggestions directed at the principal stakeholders in scholarly publishing—authors, editors, and publishers. First, it may be prudent for each author, not just the corresponding author, to sign off on a list of itemized potential AI applications at the time of manuscript submission, disallowing any use that risks scientific integrity in collaborative studies. Second, if AI tools are used in any portion of the study, each author should explicitly acknowledge what AI tools were used for which portions of the submitted manuscript^11,12^. For instance, language assistance for grammar may justify a brief acknowledgment, whereas generation of scientific content, references, figures, or data analysis warrants more detailed disclosure. Third, publishers without a centralized AI policy should consider adopting 1 set of guidelines applied uniformly across all their journals or clearly state on their author-facing website that policies for AI disclosure vary among the journals supported by the publisher. Journals could likewise place AI guidance in a dedicated IFA section. Finally, peer reviewers should be forbidden from uploading manuscripts under review into publicly accessible AI tools.

Several limitations regarding our review should be noted. While we have made some suggestions to help standardize and improve the transparency of AI use for publishers, journals, and editors, we cannot establish a causal link between more clearly outlined disclosure requirements and improved compliance with honest author responses to disclosure queries. In addition, our extraction of data from journal and publisher websites reflects a single time period (March-April 2026) that may have evolved by the time of publication. In addition, we examined only publicly accessible pages from journal and publisher websites, so submission-portal prompts and actual editorial practices may differ from stated policy. Furthermore, assessing strictness and accessibility of extracted data for this study involved some subjectivity, since these guidelines were not always stated explicitly. Finally, our sample was limited to a few English-language journals rather than a more structured and comprehensive analysis of all peer-reviewed orthopaedic journals.

While some form of AI policy for the authors is common across a sample of orthopaedic and biomedical journals, the guidelines an author encounters vary substantially. Domains that can directly impact scientific integrity and clinical practice, such as AI-assisted manipulation of data, statistical outputs, fabricated references, and undisclosed image alterations, are currently not consistently queried in detail at manuscript submission. As technology evolves, journals should test and adopt AI-detection tools that are both sensitive and specific to screen all submissions before peer-review. Greater standardization and clearer, harm-prevention guidance, particularly around fabricated references, nonverified AI data analysis, and image manipulation—without entirely banning the use of AI tools by the authors—can preserve scientific integrity while allowing the authors to harness AI tools such as those that improve grammar and comprehension.

## Funding

No funding was received for this study.

## Conflicts of Interest

The authors have no relevant financial or non-financial conflicts of interest to disclose.

## Ethics Approval

As our observations used publicly available information and did not involve human subjects, it was exempt from IRB approval.

## AI Use Disclosure

The authors used a large language model (Claude Opus 4.7, Anthropic) for proofreading and editorial assistance during manuscript preparation. All study design, data extraction, analysis, interpretation, and final content decisions were made by the authors, who verified every reference and take full responsibility for this manuscript.

## Race and Ethnicity

Race and ethnicity data are not included in the manuscript as these data were not included in the available raw data.

## Appendix

Supporting material provided by the authors is posted with the online version of this article as a data supplement at jbjs.org (https://links.lww.com/JBJSOA/B322). This content was not copyedited or verified by JBJS.
